# Metabolomics and Transcriptomics Reveal the Effects of Fermented *Lycium barbarum* (Goji) Berry Residue on Muscle Nutrition and Flavor Quality in Fattening Tan Sheep

**DOI:** 10.3390/metabo16010039

**Published:** 2026-01-01

**Authors:** Cong Zhan, Meng Li, Dan Li, Pan Li, Qiming Zhang, Mirou Wu, Guowei Zhong, Xiaochun Xu

**Affiliations:** Collaborative Innovation Center for Food Production and Safety, School of Biological Science & Engineering, North Minzu University, Yinchuan 750021, China; zhancong0301@163.com (C.Z.); 13289592120@163.com (M.L.); ld24953000473@163.com (D.L.); leepanan@163.com (P.L.); zqm20020806@163.com (Q.Z.); mirouwu0621@163.com (M.W.); 15856016176@163.com (G.Z.)

**Keywords:** fermented *Lycium barbarum* (Goji) berry residue, meat quality, muscle metabolites and flavors, lambs, amino acid and lipid metabolism

## Abstract

Background/Objectives: In the context of increasing consumer demand for high-quality meat, this study aimed to evaluate the effects of 4% fermented goji berry residue supplementation on meat quality and flavor characteristics in finishing Tan sheep. Methods: Thirty-six male lambs were randomly assigned to a control and FGB group and fed for 68 days. Results: FGB supplementation significantly enhanced *Longissimus Dorsi* (LD) brightness (L*), redness (a*), and crude protein content, while reducing crude fat (*p* < 0.05). Amino acid analysis revealed significant increases in lysine, methionine, histidine, glycine, proline, arginine, cysteine, and total sweet-tasting amino acids in the FGB group (*p* < 0.05). Lactate and inosine monophosphate (IMP) levels were significantly elevated, whereas hypoxanthine levels decreased (*p* < 0.05). Metabolomics identified 189 metabolites, with 12 differentially expressed, mainly enriched in butanoate metabolism, glycolysis/gluconeogenesis, PI3K-Akt, and HIF-1 signaling pathways. Transcriptomics revealed 382 differentially expressed genes, including key regulators of lipid metabolism (*FOXO1*, *SLC2A4*, *LPIN1*, *IGF1*, *SPP1*) and amino acid metabolism (*COL3A1*, *GLUL*, *PSMC1*). Conclusions: Fermented goji residue altered amino acid and lipid metabolism in the LD muscle of Tan sheep, affecting meat quality and flavor traits. However, effects on color (L*, a*, b*), protein content, and shear force varied across the four muscles studied, indicating that responses to supplementation are muscle-specific. These findings offer a sustainable strategy for improving meat quality and provide insights into the molecular mechanisms underlying flavor development in ruminants.

## 1. Introduction

Ningxia Tan sheep mutton is highly regarded for its tenderness, juiciness, lack of odor, and rich nutritional profile, making it popular among consumers [1,2]. However, intensive farming practices, which rely heavily on concentrates, lead to excessive fat accumulation, negatively affecting meat quality and growth rates, and often resulting in poor survival rates. Moreover, despite the growing demand for Tan sheep meat, its quality remains compromised due to a shortage of high-quality feed [3]. Key meat quality parameters—such as pH, color, and flavor—play a crucial role in consumer purchasing decisions and market performance [4]. These traits are influenced by factors like feeding regimens and dietary composition [5,6]. Therefore, developing effective dietary strategies to improve meat quality is critical to meet consumer expectations.

The sensory quality of lamb meat, including its aroma and taste, significantly impacts its commercial value. Amino acids not only enhance flavor but also act as precursors to volatile compounds, while fatty acids contribute to overall flavor, tenderness, and juiciness [7]. Among amino acids, glutamic acid and alanine intensify umami perception, while arginine is linked to bitterness [8]. Exogenous glutamine supplementation can increase aldehyde concentrations, further enhancing meat aroma [9]. Non-essential amino acids are known to influence flavor complexity, and amino acid metabolic pathways in Tibetan sheep contribute to the unique flavor characteristics of their meat, as suggested by proteomic analysis [10]. Additionally, the modulation of gut microbiota in other animals has been shown to enhance flavor profiles, underscoring the importance of amino acid composition in flavor development [11]. Both amino acid metabolic pathways and fatty acid oxidation are crucial for flavor development in meat, with linoleic acid oxidation generating compounds responsible for “fatty” and “roasted meat” aromas [12]. Aldehydes, alcohols, ketones, and furans are essential flavor compounds that contribute to a wide range of meat aromas [12,13]. Consequently, both fatty acid and amino acid composition are critical nutritional determinants influencing lamb meat’s overall quality attributes [7]. Additionally, flavor precursor synthesis is regulated by various metabolic enzymes and host genes. For example, the transcription factor *FOXO1* influences fat metabolism by interacting with multiple signaling pathways [14], while *LPIN1* plays a key role in lipid storage and breakdown [15]. *IGF1* regulates lipid metabolism through its receptor *IGF1R*, impacting lipogenesis and lipid accumulation [16]. Furthermore, enzymes like glutamine synthetase (GLUL) play a vital role in amino acid accumulation within muscle tissue [17]. Despite the link between gene expression and meat flavor, targeted regulation of genes involved in flavor precursor metabolism, particularly in Tan sheep, remains underexplored.

*Lycium barbarum* (*Goji*), a member of the Solanaceae family and predominantly cultivated in the Ningxia Autonomous Region of China, is a plant that offers both medicinal and nutritional benefits. It is rich in bioactive compounds such as polysaccharides, flavonoids, alkaloids, and betaine, all contributing to animal health and nutrition [18,19,20]. Fermentation of Goji seed meal can generate volatile compounds that enhance meat flavor [21], while its flavonoids and polysaccharides have been shown to improve antioxidant capacity, delay lipid oxidation, and preserve meat quality. Agricultural by-products, such as Goji berry residues, are often discarded, leading to environmental waste. However, when fermented, these residues can improve livestock health and meat quality [22]. Recent studies suggest that microbial fermentation of agricultural by-products can enhance their nutritional value by degrading anti-nutritional factors and improving protein digestibility [23]. For example, the inoculation of *Lactobacillus plantarum* in corn stover silage improves fiber degradation and increases lactic acid production [24]. Similarly, *Bacillus species* and *Lactic acid bacteria* have been shown to enhance the metabolic potential of carbohydrates and amino acids in feed [25], while yeast cultures can improve fiber degradation in sheep feed [26]. Given these findings, we aim to investigate the effects of fermented Goji berry residue as a dietary supplement in Tan sheep.

The aim of this study was to prepare fermented Goji berry residue by fermenting Goji berry residue with a mixed starter culture (*Lactic acid bacteria*: *Lactobacillus acidophilus*, *Enterococcus faecalis*, and *Enterococcus faecium*), yeast, and Bacillus spores and to evaluate its effects as a dietary supplement on Tan sheep. Specifically, we aimed to (i) determine its impacts on carcass traits and muscle nutritional composition (fatty acid and amino acid profiles), (ii) characterize changes in meat flavor profiles by analyzing volatile flavor compounds, and (iii) elucidate potential molecular mechanisms underlying meat quality alterations using volatile metabolomics and transcriptomics, with particular attention to intramuscular fat metabolism and muscle protein deposition.

## 2. Materials and Methods

### 2.1. Animals and Treatments

This study was approved by the Ethics Committee of North Minzu University (Approval number 2023-g2006, 20230314). Thirty-six male Tan lambs, aged 3–4 months (28.26 ± 1.03 kg), with similar body weights, were randomly assigned to two dietary treatment groups: CON and FGB. Lambs in the CON group were provided ad libitum access to a basal diet (formulation detailed in Table 1), while those in the FGB group received the same diet supplemented with 4% fermented goji berry residue for the entire 68-day trial period. Each treatment consisted of three replicates, with six lambs per replicate (*n* = 18 per group). The experimental Tan sheep and dietary raw materials were provided by Ningxia Shuomu Yanchi Tan Sheep Breeding Co., Ltd. (Wuzhong, China), and the fermented go berry residue was provided by Ningxia Lvjianyuan Biological Technology Co., Ltd. (Shizuishan, China). The changes in nutrient composition before and after fermentation of goji berry residue are shown in Table 2 when *Lactobacillus* (*Lactobacillus acidophilus*, *Enterococcus faecium*, *Enterococcus faecalis*), *Yeast*, *Bacillus*, and crude fiber–decomposition enzymes were inoculated in equal proportions. Corn, silage corn, corn straw, finishing sheep total mixed diet, and fattening feed are used to make compound feed. The fine and coarse materials were mixed and fed to Tan sheep according to the proportion of the experimental design. The basal diet was formulated in accordance with the “Feeding Standard for Meat Sheep” (NY/T 816-2021) and was further adjusted based on the specific production practices of the local sheep farm. The composition and nutritional specifications of the basal diet are presented in Table 2.

### 2.2. Sample and Data Collection

At the conclusion of the 68-day feeding trial, all lambs were weighed. Six lambs, selected randomly (two from each of the three pens per treatment group), were then transported approximately 10 km (roughly 30 min) to a local commercial abattoir. Upon arrival, the lambs were held in lairage for 12 h with access to water but no feed, before being slaughtered in the experimental abattoir according to EU regulations.

Following slaughter, the carcasses were chilled at 4 °C, reweighed, and the dressing percentage was calculated by determining the ratio of carcass weight to live body weight. Muscle samples taken immediately after slaughter—including *Longissimus Dorsi* (LD), *Triceps brachii* (TM), *Gluteus medius* (GM), and *Biceps femoris* (BF)—were collected from the left side of each carcass to assess meat color, pH, shear force, drip loss, and cooking loss. Additionally, approximately 100 g of LD muscle tissue from the 10th rib region of the left side was excised and stored at −20 °C for subsequent analysis of amino acid composition, inosine monophosphate content, fatty acid profile, and flavor-related compounds. Another LD sample from the 10th rib of the right side of the carcass was collected for transcriptomic and metabolomic analyses.

### 2.3. Determination of Meat Quality

Prior to measurement, the pH meter (Seven Go; Mettler Toledo, Greifensee, Switzerland) was calibrated at 25 °C using standard buffer solutions of pH 4.01 and 7.00. Automatic temperature compensation (ATC) was enabled to correct for sample temperature at 4 °C. Carcass pH was then measured in the left LD, TM, GM, and BF muscles at 45 min and 24 h postmortem by inserting the probe directly into samples maintained at 4 °C. After triplicate pH measurements, the samples were sequentially analyzed for color, shear force, and cooking loss. Meat color coordinates (L*, a*, b*) were recorded after 60 min of blooming using a chromometer (CR-410; Konica Minolta, Japan) in the CIELAB color space with a D65 illuminant and a 10° observation angle. Triplicate measurements were taken for each sample.

Tenderness (shear force) and cooking loss were evaluated following the method outlined. Samples were cooked in sealed plastic bags within an 80 °C water bath until the internal temperature of each sample reached 70 °C. The core temperature was monitored using a type-T thermocouple probe inserted into the geometric center of each sample, with continuous temperature readings recorded to ensure precise cooking termination. All samples were processed in a single batch to minimize variation between batches. Importantly, only one cooking batch was used to eliminate any potential batch-related effects. Sample weights were recorded both before (W_1_) and after (W_2_) thermal processing. Sample weights were measured prior to (W_1_) and following (W_2_) thermal processing. Cooking loss percentage was computed according to the expression: [(W_1_ − W_2_)/W_1_] × 100. Post-cooking specimens were cut in a direction parallel to the muscle fibers into standardized parallelepipeds (3 cm × 1 cm × 1 cm; mean weight 3.2 ± 0.2 g) from the LD, TM, GM, and BF muscles. Shear force was measured on each block using a texture analyzer equipped with a strain-gauge load cell (C-LM3B, Northeast Agricultural University, Harbin, China). A minimum of 8 measurements were performed per individual sample to ensure technical precision.

### 2.4. Muscle Chemical Analysis

Approximately 50 g samples of *longissimus dorsi* (LD), *Triceps brachii* (TM), *Gluteus medius* (GM), and *Biceps femoris* (BF) muscles were collected and weighed 48 h postmortem for proximate composition analysis. The nutritional profiles of the samples were determined using standardized methods outlined by the Association of Official Analytical Chemists (AOAC International, 2005). Moisture content was measured by oven-drying the samples at 105 °C to constant weight (AOAC Method 934.01). Crude protein content was calculated as total nitrogen × 6.25, with nitrogen determined using the Kjeldahl digestion and distillation procedure (AOAC Method 984.13). Crude fat content was quantified by Soxhlet extraction using petroleum ether as the solvent (AOAC Method 960.39). Ash content was determined by incinerating the samples in a muffle furnace at 550 °C until constant weight was achieved (AOAC Method 942.05).

### 2.5. Muscle Inosinic Acid Content Analysis

Five grams of fresh *Longissimus dorsi* muscle were homogenized in 25 mL of 5% perchloric acid, followed by centrifugation at 10,000× *g* for 10 min at 4 °C to obtain the supernatant. The residue was re-extracted with another 25 mL of 5% perchloric acid and centrifuged again, and the combined supernatants were neutralized to pH 6.5 with 1 M potassium hydroxide solution. The neutralized extract was then diluted to a final volume of 100 mL with ultrapure water and filtered through a 0.45 μm membrane filter prior to high-performance liquid chromatography (HPLC) analysis. The HPLC system (e.g., Agilent 1260 series or equivalent) was equipped with a reversed-phase C18 column (250 mm × 4.6 mm, 5 μm particle size) maintained at 25 °C. The mobile phase consisted of 0.05 M phosphate buffer (pH 6.5) containing 1% methanol, delivered isocratically at a flow rate of 1.0 mL/min. Detection was performed using a UV detector at 254 nm. Inosinic acid (IMP) was identified and quantified by comparison with authentic standards, with the content expressed as mg/100 g muscle tissue.

### 2.6. Muscle Amino Acid Composition Analysis

An appropriate amount of lyophilized muscle sample (typically containing 5–10 mg protein) was weighed and placed into a 50 mL hydrolysis tube, to which 20 mL of 6 M hydrochloric acid (containing 0.1% phenol to protect tyrosine) was added. The tube was then purged with nitrogen, sealed, and hydrolyzed at 110 °C for 22 h in an electric blast drying oven. After hydrolysis, the tube was removed and cooled to room temperature, and the hydrolysate was transferred to a 25 mL volumetric flask and adjusted to volume with ultrapure water. Subsequently, 50 μL of the hydrolysate was accurately transferred to a 5 mL centrifuge tube and evaporated to dryness in a vacuum drying oven at 60 °C for 2 h. The dried residue was redissolved in 1.0 mL of ultrapure water and thoroughly mixed. The solution was then filtered through a 0.45 μm membrane filter into an autosampler vial prior to derivatization and analysis by high-performance liquid chromatography (HPLC) using an Agilent 1260 system equipped with an AdvanceBio AAA reversed-phase column (4.6 mm × 100 mm, 2.7 μm particle size) maintained at 40 °C. Automated pre-column derivatization with o-phthalaldehyde (OPA) for primary amino acids and 9-fluorenylmethyl chloroformate (FMOC) for secondary amino acids was performed in the autosampler. The mobile phase consisted of (A) 40 mM sodium phosphate buffer (pH 7.8) and (B) acetonitrile/methanol/water (45/45/10, *v*/*v*/*v*), with a gradient elution program and a flow rate of 1.0–1.5 mL/min. Detection was carried out using a fluorescence detector (excitation 340 nm, emission 450 nm for OPA derivatives; excitation 266 nm, emission 305 nm for FMOC derivatives) or a UV detector at 338 nm [27].

### 2.7. Fatty Acid Analysis

*Longissimus dorsi* (LD) muscle samples were thawed and homogenized, and 1.0 g aliquots were weighed into 10 mL screw-capped tubes for fatty acid analysis. Tridecanoic acid (C13:0) was added as an internal standard. To each tube, 0.7 mL of 10 mol/L potassium hydroxide and 5.3 mL of anhydrous methanol were added. The tubes were shaken for 5 s every 20 min while incubating in a 55 °C water bath for 1.5 h to achieve saponification. After cooling to room temperature with tap water, 0.58 mL of 12 mol/L sulfuric acid was added, and the incubation steps were repeated to complete methylation. Subsequently, 3 mL of n-hexane was added, and the mixture was vigorously vortexed, transferred to a centrifuge tube, and centrifuged at 3000 rpm for 5 min. The upper hexane layer containing fatty acid methyl esters (FAMEs) was collected, filtered through a 0.22 μm organic phase membrane into a sample vial, and concentrated from 2 mL to approximately 1.5 mL at 45 °C under a gentle nitrogen stream if necessary. FAMEs were analyzed using a Shimadzu GC-2010 Plus gas chromatograph equipped with a flame ionization detector and an SP™-2560 capillary column (100 m × 0.25 mm i.d., 0.20 μm film thickness; Supelco, Bellefonte, PA, USA). The injector and detector temperatures were both set at 250 °C. Nitrogen was used as the carrier gas at a constant flow rate of 1.0 mL/min, with a split injection mode and an injection volume of 1.0 μL. The oven temperature program was as follows: initial temperature of 140 °C held for 5 min, increased to 240 °C at 4 °C/min, and held at 240 °C for 20 min. Individual fatty acids were identified by comparison with known FAME standards (37-component FAME mixture; Supelco) and quantified using peak area normalization with the internal standard for absolute content or relative percentage calculation [7].

### 2.8. Volatile Flavor Components (VOCs) Analysis

For the determination of volatile flavor compounds in the *longissimus dorsi* muscle of Tan sheep, approximately 10 g of minced meat sample was weighed and placed into a 20 mL headspace vial, to which 20% (*w*/*w*) sodium chloride was added to enhance the release of volatiles. The vial was sealed with a PTFE/silicone septum and equilibrated at 80 °C for 1 h in a thermostatic water bath with occasional stirring using a glass rod. Volatile compounds were extracted using headspace solid-phase microextraction (HS-SPME) with a preconditioned 50/30 μm DVB/CAR/PDMS fiber (Supelco, Bellefonte, PA, USA). The aged fiber was inserted into the headspace through the septum and exposed for 40 min at 80 °C to adsorb the volatiles. Subsequently, the fiber was withdrawn and immediately inserted into the GC injector port for thermal desorption at 250 °C for 5 min in splitless mode. Analysis was performed on an Agilent 7890B gas chromatograph coupled to a 5977A mass spectrometer (Agilent Technologies, Santa Clara, CA, USA) equipped with an HP-5MS capillary column (30 m × 0.25 mm i.d., 0.25 μm film thickness). Helium was used as the carrier gas at a constant flow rate of 1.0 mL/min. The oven temperature program was as follows: initial temperature of 50 °C held for 1 min, increased to 220 °C at 3.5 °C/min, and held at 220 °C for 20 min. The injector temperature was maintained at 250 °C. Mass spectra were acquired in electron ionization mode at 70 eV with a solvent delay of 2 min, scanning in the m/z range of 50–550. Compounds were qualitatively identified by comparing their mass spectra and retention indices with those in the NIST 14 and Wiley 7n mass spectral libraries, retaining only those with match factors and reverse match factors greater than 800 (maximum 1000). Relative abundances were calculated using the peak area normalization method and expressed as percentages of the total chromatographic peak area.

### 2.9. Metabolome Analysis

The LD muscle of Tan sheep was used for volatile compound and metabolite profiling. A 2 g tissue sample and 1.125 g sodium chloride were mixed in a 10 mL vial, sealed, and subjected to solid-phase microextraction (SPME) using a Supelco (Bellefonte, PA, USA) DVB/CAR/PDMS fiber preconditioned at 250 °C. After extraction at 60 °C for 40 min, the fiber was desorbed at 250 °C for 30 s. Analyte separation was performed on an HP-5MS column with helium as the carrier gas, using a 1 μL injection. Mass spectrometry was performed with electron impact ionization at 70 eV, and data were acquired in full-scan mode (*m*/*z* 50–550). Peak areas were normalized and analyzed using Agilent MassHunter software Acquisition B.08.00. Multivariate statistical analyses, including principal component analysis (PCA) and orthogonal partial least squares discriminant analysis (OPLS-DA), were conducted to assess data discrimination and model reliability. Metabolites with a variable importance in projection (VIP) score > 1.0 and *p* < 0.05 were considered significantly different. Metabolite identification and annotation were performed using the KEGG, HMDB, and LIPID MAPS databases.

### 2.10. RNA Extraction and Transcriptome Sequencing Analysis

Total RNA was extracted from *longissimus dorsi* muscle using TRIzol Reagent, and quality was assessed with NanoDrop 2000 (Thermo Fisher Scientific, Wilmington, DE, USA) and Agilent Bioanalyzer 2100 (Agilent Technologies, Santa Clara, CA, USA). RNA libraries were constructed and sequenced on the Illumina NovaSeq 6000 platform (PE150). Raw data were filtered for adapters and low-quality reads, then aligned to the Bos taurus genome using HISAT2. Gene expression was quantified by FPKM, and differential expression was analyzed with DESeq2 (|log2FC| > 1, FDR < 0.01). Functional annotation and enrichment analyses of DEGs were performed using GO, KEGG, and clusterProfiler (version, 4.10.0). The analysis results of the Sankey Bubble Chart were generated using the CNSknowall platform (https://cnsknowall.com), a comprehensive web service for data analysis and visualization.

### 2.11. Transcriptome and Metabolome Joint Analysis

To integrate transcriptomic and metabolomic data for elucidating potential regulatory mechanisms, differentially expressed genes and differentially accumulated metabolites were subjected to joint Kyoto Encyclopedia of Genes and Genomes (KEGG) pathway analysis. Significantly co-enriched pathways were defined as those with FDR-adjusted *p* < 0.05. The number of shared pathways between DEGs and DMs was illustrated using a Venn diagram. Key enriched pathways were visualized in a bubble chart displaying enrichment ratio, impact value, and adjusted *p*-value. Gene–metabolite pathway associations were further depicted using a Sankey diagram generated within the same platform [28].

### 2.12. Statistical Analysis

Data are presented as means ± standard error of the mean (SEM) and were tested for normality (Shapiro–Wilk’s W test) and homogeneity of variance (Levene’s test). Data, including meat quality traits, muscle chemical composition, amino acid content, and volatile flavor compounds, were analyzed using a linear mixed model in SPSS 25.0 (SPSS Inc., Chicago, IL, USA). The model was specified as follows:

y_ij_ = μ + D_i_ + P_j_ + ε_ij_,

where y_ij_ is the observed value of the dependent variable; μ is the overall mean; D_i_ is the fixed effect of the ith dietary treatment (i = control, fermented *Lycium barbarum* residue); P_j_ is the random effect of the jth pen (j = 1 to 6); and ε_ij_ is the random residual error. Growth performance and meat quality were analyzed using the pen as the experimental unit [29]. Differences among means were considered statistically significant at *p* < 0.05 and considered to exhibit a trend toward significance when 0.05 ≤ *p* ≤ 0.10.

## 3. Results

### 3.1. Meat Quality of LD, TM, GM, and BF Muscles

Table 3 presents the effects of dietary supplementation with fermented Goji berry pomace (FGB) on the main quality indicators of meat from four major muscles of Tan sheep—*Longissimus dorsi* (LD), *Triceps brachii* (TM), *Gluteus medius* (GM), and *Biceps femoris* (BF). No significant differences were observed in pH at 45 min postmortem (pH_45min_) across treatment groups for any of the muscles. At 24 h postmortem, pH values remained stable for all muscles, with no significant differences observed, indicating that FGB supplementation did not affect the long-term pH stability of the meat.

FGB supplementation significantly altered meat color parameters across muscles. Specifically, lightness (L*) was significantly increased in LD, TM, and GM muscles (*p* < 0.001) compared to controls. Redness (a*) showed a significant increase only in the LD muscle (*p* < 0.001), suggesting that FGB has a muscle-specific effect on color attributes.

Water-holding capacity, as indicated by cooking loss and drip loss, was preserved with FGB supplementation, with no significant differences observed between treatment groups (*p* > 0.05). Shear force, a measure of meat tenderness, significantly increased only in the GM muscle (*p* = 0.010), while no significant changes were noted in other muscles. This suggests that the effect of FGB on tenderness is muscle-dependent.

FGB supplementation did not significantly alter moisture or ash content in the LD, TM, GM, or BF muscles (*p* ≥ 0.05; Table 4). However, it significantly increased crude protein content in the GM, LD, and BF muscles, while simultaneously causing significant reductions in crude fat content across all muscles (TM, GM, LD, and BF) (*p* < 0.001). These findings suggest that FGB supplementation enhances protein content while reducing fat levels, which may contribute to improved nutritional quality of the meat.

### 3.2. Amino Acid Composition of LD Muscle

In the LD muscle, FGB supplementation significantly increased several flavor-related amino acids, including lysine, methionine, glycine, proline, arginine, and cysteine (*p* < 0.05; Table 5), while essential amino acids and total amino acids exhibited increasing trends (0.05 < *p* < 0.10). These changes are consistent with improved flavor precursor availability.

### 3.3. Meat Flavor Compounds Contents of LD Muscle

This study investigated the flavor compound profile in the LD of Tan sheep and examined the effect of incorporating fermented goji pomace into the diet on its flavor composition (Table 6). The findings demonstrate that the inclusion of fermented goji pomace notably enhanced the concentrations of lactic acid and inosine monophosphate (IMP) in the LD (*p* < 0.05), while simultaneously causing a significant reduction in hypoxanthine content (*p* < 0.001).

### 3.4. Fatty Acid Composition of LD Muscle

Dietary supplementation with fermented goji pomace (FGB) significantly altered the fatty acid profile of Tan sheep meat (Table 7). In comparison with the control group (CON), the FGB treatment tended to reduce the total saturated fatty acid (SFA) concentration (0.05 < *p* < 0.1), primarily attributable to significant decreases observed in myristic (C14:0) and palmitic (C16:0) acids (*p* < 0.05). The total monounsaturated fatty acid (MUFA) fraction was markedly lowered in the FGB group (*p* < 0.05), a result largely driven by diminished levels of heptadecenoic (C17:1n7), cis-9-octadecenoic (oleic acid, C18:1n9 cis), and trans-9-octadecenoic (C18:1n9 trans) acids, whereas vaccenic acid (C18:1n7) exhibited a significant increase (*p* < 0.05).

### 3.5. Volatile Compounds of LD Muscle

**Figure 1:**
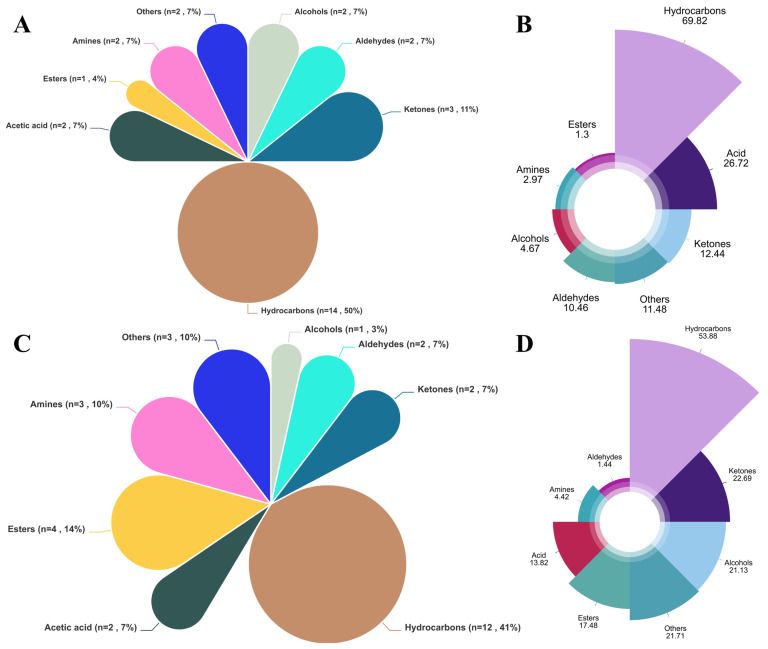
Influence of dietary supplementation with fermented goji berry pomace subjected to fermentation on the composition and relative abundance of volatile compounds in the *Longissimus dorsi* muscle of Tan sheep. (**A**) Proportion of volatile compound classes detected in the *Longissimus dorsi* muscle of animals from the control group. (**B**) Relative contribution (%) of each volatile compound class to the total volatile profile in the control group. (**C**) Proportion of volatile compound classes identified in the *Longissimus dorsi* muscle of animals receiving the fermented goji berry pomace (FGB) treatment. (**D**) Relative contribution (%) of each volatile compound class to the total volatile profile in the FGB-supplemented (FGB) group.

The inclusion of fermented wolfberry pomace in the diet resulted in changes to the types of volatile flavor compounds (Figure 1A and Table 8). A total of 44 volatile compounds (VOCs) were identified in the *Longissimus dorsi* muscles of both the CON and FGB groups, which were classified into 8 categories: alcohols, aldehydes, ketones, hydrocarbons, acids, esters, amines, and others. The CON group exhibited 28 volatile compounds, while the FGB group detected 29. The relative contents of alcohols and aldehydes in the FGB group differed significantly from those in the CON group (*p* < 0.05). Specifically, the relative levels of 1-butanol and carbon disulfide were significantly higher in the FGB group than in the CON group, while those of phenylacetaldehyde, decane, and 1,3,5-cycloheptatriene were significantly reduced (*p* < 0.05).

### 3.6. Metabolome of LD Muscle

**Figure 2:**
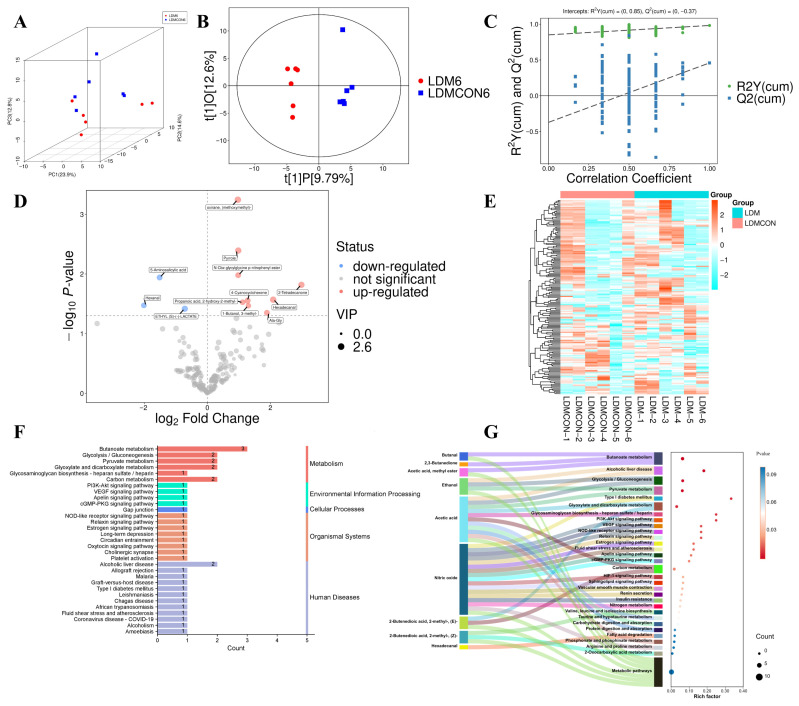
Off-target metabolomic analysis of the *Longissimus dorsi* muscle in Tan sheep. (**A**) 3D score scatter plot of the PCA model comparing groups LDMCON6 vs. LDM6. (**B**) Score scatter plot of the OPLS-DA model comparing groups LDMCON6 vs. LDM6. (**C**) Permutation test plot validating the OPLS-DA model for groups LDMCON6 vs. LDM6. (**D**) Volcano plot displaying the differential metabolites between LDMCON6 and LDM6. (**E**) Heatmap showing hierarchical clustering analysis across all groups. (**F**) Histogram illustrating KEGG pathway enrichment in the LDMCON6 group vs. LDM6. (**G**) KEGG Sankey bubble plot comparing the LDMCON6 group with LDM6.

Untargeted metabolomic profiling using GC-TOFMS revealed that dietary supplementation with fermented Goji berry pomace (FGB) induced significant changes in the metabolite composition of the *Longissimus dorsi* (LD) muscle in Tan sheep. Both principal component analysis (PCA) and orthogonal partial least squares-discriminant analysis (OPLS-DA) showed clear separation in the metabolic profiles between the FGB-supplemented group (LDM6) and the control group (LDMCON) (Figure 2A,B). The robustness of the OPLS-DA model was further validated through permutation tests, resulting in a cumulative R^2^Y value of 0.85 (Figure 2C), confirming the model’s predictive ability.

**Figure 3:**
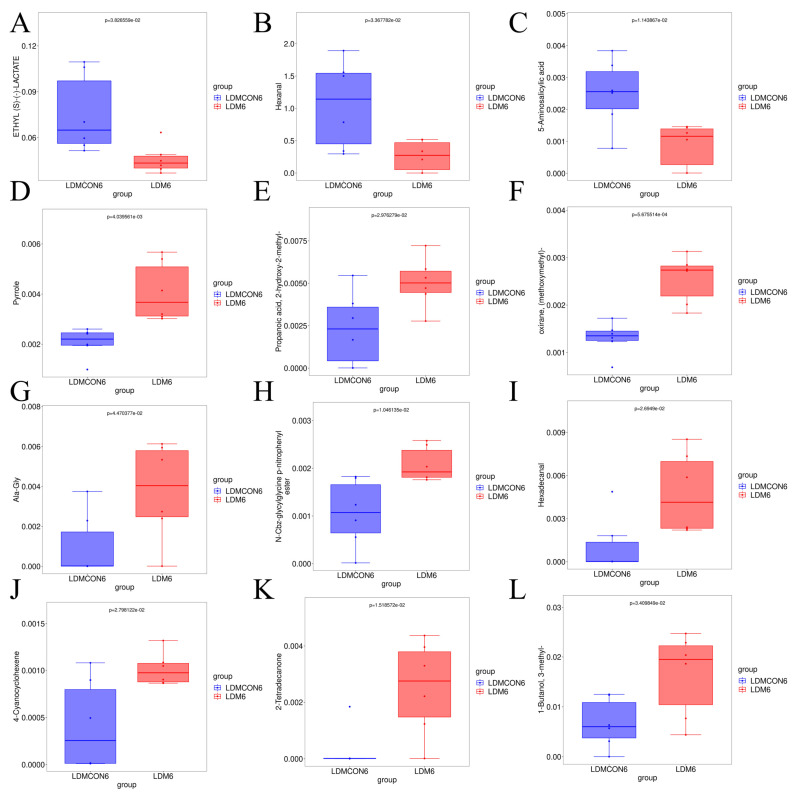
Boxplot of 12 differentially identified metabolites by metabolomics in the LDM and LDMCON groups. (**A**–**C**) downregulated; (**D**–**L**) upregulated.

Out of the 189 metabolites detected, 12 were identified as significantly differentially expressed metabolites (DEMs; VIP > 1, *p* < 0.05), including nine upregulated and three downregulated compounds (Figure 2D, Figure 3). Hierarchical clustering analysis of these DEMs revealed distinct expression patterns between the FGB-supplemented and control groups (Figure 2E), highlighting the effect of FGB supplementation on the metabolomic profile of the LD muscle.

KEGG pathway enrichment analysis of the differentially accumulated metabolites showed that these metabolites were primarily involved in key metabolic and signaling pathways. Notably, these pathways included energy metabolism (e.g., butanoate metabolism, glycolysis/gluconeogenesis, pyruvate metabolism, and glyoxylate and dicarboxylate metabolism), signaling cascades (such as PI3K-Akt and HIF-1 signaling pathways), and processes related to alcoholic liver disease (Figure 2F,G). These results suggest that FGB supplementation induces metabolic shifts in LD muscle, particularly in energy production and signaling pathways, which may contribute to improved meat quality.

### 3.7. Transcriptome of LD Muscle

**Figure 4:**
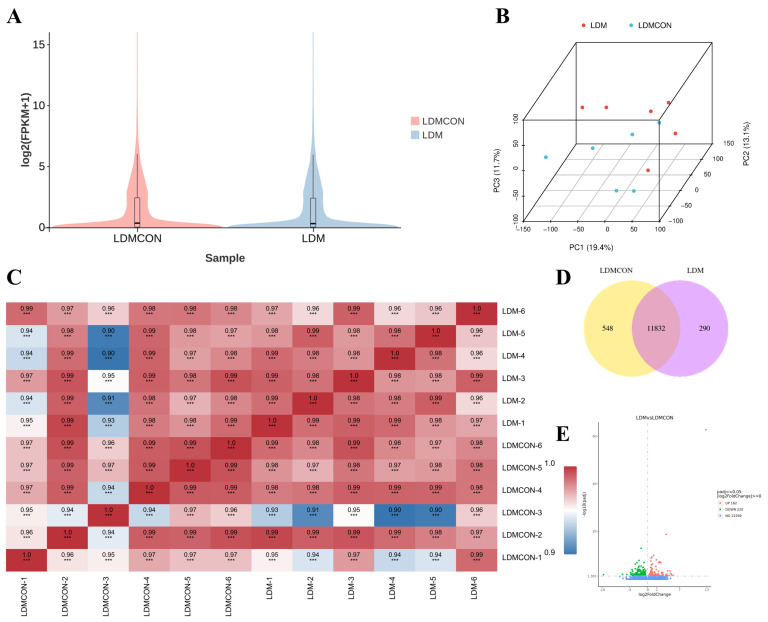
Effect of FGB supplementation on the transcriptome profile of *Longissimus dorsi* (LD) tissues in Tan sheep. (**A**) Violin plots of FPKM (Fragments Per Kilobase of transcript per Million mapped reads) values for all samples. (**B**) Three-dimensional PCA plot illustrating sample distribution across groups. (**C**) Heatmap showing correlation patterns among all samples (*** indicates *p* < 0.001). (**D**) Venn diagram depicting the overlap of differentially expressed genes (DEGs) between the LDM and LDMCON groups. (**E**) Volcano plot highlighting the differentially expressed genes between the LDM and LDMCON groups.

RNA-seq analysis of the *Longissimus dorsi* (LD) muscle from Tan sheep (6 FGB-supplemented vs. 6 control animals) provided high-quality data, with clean data ≥ 6.1 GB per sample, Q30 > 94.98%, and 88.3–89.67% of clean reads successfully mapping to the reference genome (Table S2). Principal component analysis (PCA) (Figure 4B) and correlation analysis (Figure 4C) revealed clear differences in the transcriptomic profiles between the FGB-supplemented and control groups. In total, 382 differentially expressed genes (DEGs) were identified, with 162 genes upregulated and 220 genes downregulated (Figure 4E, Table S3).

**Figure 5:**
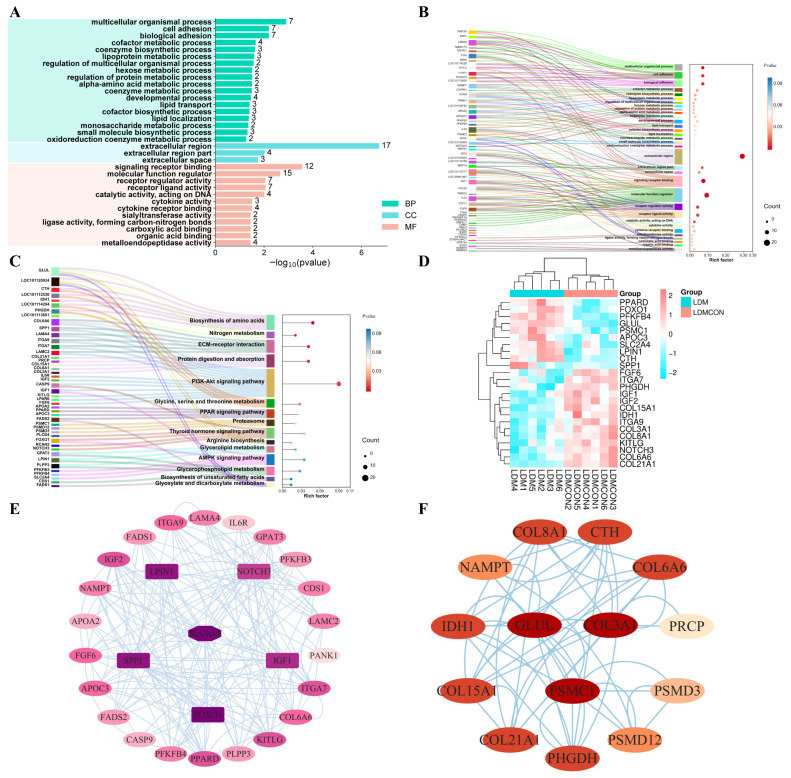
Enrichment analysis and PPI (Protein–Protein Interaction) network construction of transcriptome profiles from *Longissimus dorsi* (LD) tissues of Tan sheep following FGB supplementation. (**A**) Bar graph representing GO (Gene Ontology) enrichment analysis. (**B**) Sankey bubble plot illustrating GO enrichment analysis. (**C**) Sankey bubble plot for KEGG pathway enrichment analysis. (**D**) Hierarchical clustering heatmap of differentially expressed genes (DEGs). (**E**) PPI network related to lipid metabolism. (**F**) PPI network related to amino acid synthesis.

Gene Ontology (GO) enrichment analysis of these DEGs revealed significant associations with 35 cellular components, 144 molecular functions, and 193 biological processes (Table S4). Notably, these genes were enriched in pathways related to coenzyme/amino acid biosynthesis, hexose/monosaccharide metabolism, and lipid transport/localization (Figure 5B), highlighting the importance of these processes in muscle metabolism and development.

KEGG pathway analysis further identified key metabolic and signaling pathways critical for meat quality. These included pathways associated with amino acid biosynthesis, nitrogen metabolism, glycerol (phospho)lipid metabolism, unsaturated fatty acid biosynthesis, and PPAR/PI3K-Akt/AMPK signaling, as well as ECM–receptor interactions and protein digestion/absorption (Figure 5C, Table S5). Of particular interest, lipid metabolism and amino acid synthesis pathways were identified as central areas of regulation for further investigation (Table S6).

To visualize the protein–protein interaction (PPI) network, STRING data were imported into Cytoscape (version 3.9.1). Genes within the network were ranked based on degree values, and core genes were identified, highlighted in darker colors (Figure 5E,F). Genes such as *FOXO1*, *SLC2A4*, *LPIN1*, *IGF1*, *SPP1*, *COL3A1*, *GLUL*, and *PSMC1* were located at the core of both PPI networks, suggesting their pivotal role in regulating muscle function and development. A hierarchical clustering heatmap was created based on the top-ranked genes from both PPI networks (Figure 5D). The heatmap revealed that genes such as *PPARD*, *FOXO1*, *PFKFB4*, *GLUL*, *PSMC1*, *APOC3*, *SLC2A4*, *LPIN1*, *CTH*, and *SPP1* were highly expressed in the LD muscle, whereas genes like *FGF6*, *ITGA7*, *PHGDH*, *IGF1*, *IGF2*, *COL15A1*, *IDH1*, *ITGA9*, *COL3A1*, *COL8A1*, *KITLG*, *NOTCH3*, *COL6A6*, and *COL21A1* showed low expression levels, further illustrating the transcriptional divergence between treatment groups.

### 3.8. Joint Transcription and Metabolomics Analysis

**Figure 6:**
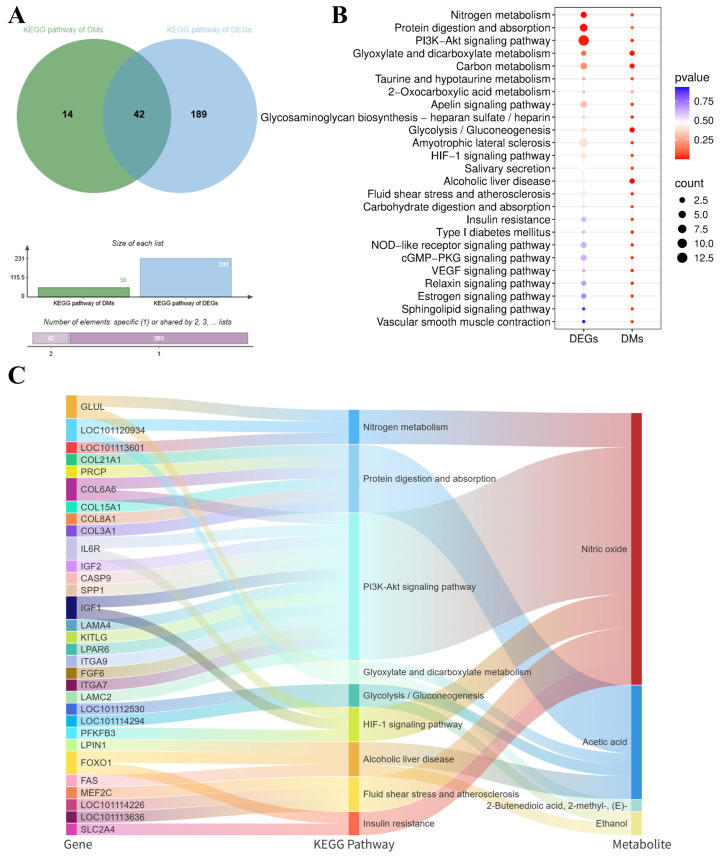
Combined transcriptomics and metabolomics analysis of Tan sheep LD tissue by FGB supplementation. (**A**) Venn plots of KEGG enrichment analysis results for transcriptome and metabolome. (**B**) KEGG bubble map of transcriptome and metabolome co-enrichment. (**C**) Gene–KEGG pathway–metabolite Sankey diagrams.

Transcriptomic-metabolomic integration revealed regulatory mechanisms underlying meat quality/flavor. KEGG co-enrichment analysis identified 42 pathways shared by DEGs and metabolites (Figure 6A). Key enriched pathways were visualized via a bubble chart, with gene–metabolite pathway relationships mapped in a Sankey diagram (Figure 6B).

The key KEGG pathways identified in this study include nitrogen metabolism, protein digestion and absorption, the PI3K-Akt signaling pathway, glyoxylate and dicarboxylate metabolism, glycolysis/gluconeogenesis, the HIF-1 signaling pathway, alcoholic liver disease, fluid shear stress and atherosclerosis, and insulin resistance. These pathways contain a total of 31 differentially expressed genes, including *GLUL*, *COL21A1*, *COL6A6*, *COL15A1*, *COL8A1*, *COL3A1*, *IL6R*, *IGF2*, *SPP1*, *IGF1*, *ITGA7*, *PFKFB3*, *FOXO1*, *FAS* and *SLC2A4*, which are strongly associated with key differential metabolites involved in important biological functions, such as nitric oxide, acetic acid, 2-butenedioic acid (2-methyl-, (E)-), and ethanol.

## 4. Discussion

The residual active components of goji berry residue (such as polysaccharides and polyphenols) can form complexes with feed proteins. Previous studies have shown that nanocrystals formed by self-assembly of goji leaf extract and whey protein can enhance the bioavailability of polyphenols and strengthen their antioxidant effects. These complexes may help protect muscle cell membrane integrity, potentially attenuate postmortem stress responses, and thereby improve meat tenderness; however, this proposed mechanism requires further experimental validation [31]. Jiale Liao discovered that fermented goji berry residue supplementation could have a significant impact on improving the average daily gain, feed efficiency, and nutrient digestibility in Tan sheep, but it did not investigate its implications on muscle amino acid and fatty acid compositions and volatile compounds. Thus, it is still unclear what effect fermented goji berry residue has on the nutritional properties and flavor quality of Tan sheep muscle. Accordingly, the aim of the present study was to evaluate the effects of dietary inclusion of 4% fermented goji berry residue (FGB) on carcass traits and meat quality, with a particular focus on muscle fatty acid and amino acid profiles and flavor-related compounds. Using a combined RNA-seq transcriptomic and GC-TOFMS-based untargeted metabolomic approach, we further explored potential molecular mechanisms associated with changes in meat quality.

### 4.1. Impact of FGB on Meat Quality in Fattening Tan Sheep

An important visual attribute of meat that determines consumer choices of lamb is its meat color, which is measured in brightness (L*), redness (a*), and yellowness (b*). According to the research, Chinese customers are usually more interested in the meat color traits of local lamb, and the issues related to food safety are closely associated with the meat color stability [32]. To illustrate this, LD of lambs in rotating grazing (NG) had higher values of lightness (L*) and yellowness (b*), which can more likely match the preferences of consumers towards a fresh appearance [33]. This study found that FGB supplementation significantly altered the meat color parameters of various muscles, and compared to the control group, FGB significantly increased the brightness (L*) of LD, TM, and GM, while the redness (a*) was only significantly increased in LD, consistent with previous research results [7,34]. A higher degree of redness (a*) denotes more vividness of color and fresher meat, and fermented *Lycium barbarum* residue can greatly enhance the vital visual characteristics of Tan lamb meat. Variations in pH have a direct influence on the stability and oxidation status of myoglobin and, thus, the color of meat. The higher the pH, the darker the coloration of the meat, and the lower the pH, the higher the L value [35]. The pH_45min_ of the LD of the FGB group showed a downward trend, which can be one of the reasons why the L value of this muscle is high. In the processing of lamb, pH reduction can enhance the quality of meat, yet it can have an adverse effect on tenderness when it is too acidic [36]. Tenderness is a very important factor in meat quality; it indicates the structure of muscle fibers and the content of fats. This finding proposes that the effect of FGB supplementation on muscle fiber structure or collagen composition of GM can be stronger. Furthermore, pH changes control the intensity and juiciness of the meat flavor by controlling the enzyme activities and the metabolic byproducts (e.g., lactate and volatile compounds) [37]. In summary, the LD underwent a more detailed study of amino acids, flavor compounds, fatty acids, and volatile compounds to further explain the impact of fermented goji pomace supplementation on the quality and flavor of its meat in Tan sheep.

In addition, studies have shown that an increase in IMF can significantly enhance tenderness, juiciness, and flavor acceptance. Consumers rate the tenderness and flavor of lamb with high IMF (e.g., 4.4%) higher, but excessively high IMF (e.g., >4.4%) may lead to a greasy sensation and reduce acceptance [38]. Our research found that the fat content of muscles at different parts in the control group was higher than 4.4%, which may be due to the use of fattening basic feed and cage feeding methods. However, the FGB group significantly reduced the fat content in muscles at parts such as LD, which may effectively reduce the greasy sensation caused by excessive fat content, thereby improving the flavor of fattened Tan sheep and increasing consumer acceptance. Our results also showed that shear force, cooking loss, and drip loss did not change significantly, which may be because the range in which IMF content affects tenderness and juiciness is mainly between 1% and 6% [39,40]. Although our fat content results decreased, they did so compared to the control group (>6%), and the reduced fat content was close to 6%, especially in LD tissue, which is in the desirable range for consumer acceptance. Therefore, our study found that fermented goji berry residue could reduce fat content to improve flavor acceptability without adversely affecting tenderness and juiciness, maintaining the water-holding capacity of Tan sheep muscle. The mechanism by which IMF influences flavor mainly involves lipid metabolism and fatty acid derivatives. The fatty acids in IMF produce key volatile compounds during oxidation [41]. Changes in IMF are often accompanied by alterations in lipid composition, mainly in the composition and content of saturated fatty acids (SFAs), monounsaturated fatty acids (MUFAs), and polyunsaturated fatty acids (PUFAs), which may enhance flavor and the nutritional health of fatty acids [42]. Also, mutton is known to have a direct impact on the nutritional values and flavor due to the content of crude protein. In taste development, the aroma precursors of mutton (proteins and free amino acids) are closely associated with volatile organic compounds (VOCs), and proteins and their breakdown products (e.g., free amino acids and sulfur-based compounds) are major contributors to mutton flavor and aroma [43]. Our analysis revealed that fermented goji berry residue had the potential to increase the protein content of the LD muscles in fattened Tan sheep that could benefit the amino acid content and volatile compounds and improve the nutritional quality and flavor of Tan sheep muscles. Thus, we proceed to select LD on a full-scale analysis of the amino acids, flavor compounds, fatty acids, and volatile substances to further examine how fermented goji berry residue supplement affects the quality and flavor of Tan sheep meat.

From an industry perspective, these findings suggest that fermented Goji berry residue (FGB) could be developed as a functional feed ingredient for finishing Tan sheep, with the dual benefits of improving carcass/meat quality traits (e.g., reduced excessive fat deposition and improved visual appearance) and valorizing goji-processing by-products. In practical feeding systems, incorporating FGB at a fixed inclusion level (4% in the present study) may provide a feasible strategy to partially replace conventional concentrate ingredients and enhance value-added lamb production, while simultaneously reducing the environmental burden associated with the disposal of goji residues. Nevertheless, before large-scale adoption, further work is needed to evaluate production cost, fermentation scalability, batch-to-batch compositional consistency, and formulation compatibility under commercial conditions (including impacts on palatability, feed intake, and on-farm economics).

### 4.2. Impact of FGB on the Amino Acid Profiling of Longissimus Dorsi Muscle in Finishing-Phase Tan Sheep

In terms of free amino acids (FAAs), the FGB group exhibited overall higher concentrations, which are known to enhance meat flavor [44]. Although the total content of free amino acids (FAAs) was elevated in the FGB group, the relative proportions of individual free amino acids showed no substantial differences when compared to the control (CON) group. Among the essential amino acids, lysine (Lys) remained the most abundant, whereas glutamic acid (Glu), aspartic acid (Asp), and arginine (Arg) continued to dominate the non-essential amino acid fraction, in agreement with previously reported observations [45,46]. FAAs serve as important flavor precursors in lamb, contributing to bitterness, sweetness, and umami taste [47]. Glutamic acid (Glu), in particular, is recognized as a key umami compound and, along with other FAAs, is associated with salty and broth-like flavor attributes in meat [48]. In the LDM, *GLUL* expression was upregulated, and the gene was enriched in the “Alanine, aspartate, and glutamate metabolism” and “Glutamatergic synapse” pathways. *GLUL* is crucial in various physiological and pathological processes. Variants of this gene have been associated with coronary artery disease (CAD) and type 2 diabetes, influencing glutamate metabolism [49]. Thus, we can assume that the high levels of GLUL found in the FGB group lambs could be increasing glutamate production, but their exact contribution to glutamate production in the intramuscular lambs is subject to further research. It is important to note that aspartic acid (Asp) levels were much higher in the FGB group than in the CON group. Asp is reported to add umami taste, particularly when supplemented by sodium salts acting the same way as monosodium glutamate (MSG) [50]. Tasting of amino acid bitter-tasting (Arg, His, Ile, Leu, Met, and Val) found no significant changes in overall content between treatment groups. Despite the overall similarity in the proportional distribution of individual free amino acids between the control (CON) and FGB groups, the FGB treatment led to substantially higher absolute concentrations of sweet-tasting amino acids, most notably glycine (Gly) and threonine (Thr). This elevation consequently produced a marked increase in the total concentration of sweet-contributing free amino acids (FAAs). KEGG pathway enrichment analysis further revealed that differentially expressed genes were significantly associated with the glycine, serine, and threonine metabolism pathway, suggesting enhanced metabolic flux through this route in the FGB-supplemented animals. *CTH* had high levels of expression in the LDM, but *PHGDH* was not expressed. Earlier studies show that both *PHGDH* and *CTH* play a role in the serine–glycine–one-carbon (SGOC) metabolism in macrophages, and both genes are upregulated together due to HIV infection to impair the amino acid metabolism [51]. Moreover, PHGDH, the rate-limiting enzyme in serine production, is subject to the activity regulation by the acetylation changes triggered by serine starvation [52]. Thus, fermented goji pomace is probably capable of modulating Gly, Ser, and Thr levels in Tan sheep *Longissimus dorsi* through the control of the gene expression patterns of the major glycine–serine–threonine metabolic pathway.

The perceived sweetness can also be increased by the synergistic effect of the sweet amino acid and other flavor compounds, such as inosine monophosphate (IMP) [53]. The FGB group showed much higher levels of sweet-tasting amino acids, as well as inosine monophosphate (IMP), compared to controls, which shows that the joint action of increased umami and sweet amino acids can be used to collectively influence the sensation of flavor. This enhancement probably leads to high sensory attributes and the quality of meat in general. Moreover, improving the protein deposition, in particular, the effectiveness of muscle protein accretion, is one of the major goals in animal husbandry [54]. Past research indicates that amino acids like arginine (Arg), histidine (His), and serine (Ser) contribute to protein accumulation [55,56,57], which is in line with our results about amino acid and protein content. On the basis of this evidence, we postulate that fermented goji pomace dietary supplementation could increase the levels of Arg, His, and Ser in the *Longissimus dorsi*, leading to protein synthesis.

### 4.3. Impact of FGB on Flavor Compound Content in Longissimus Dorsi Muscle of Finishing Tan Sheep

In postmortem glycolysis in meat, the sequential degradation of ATP occurs with the resultant formation of nucleotide-related flavor compounds. ATP is broken down by different muscle enzymes into ADP, AMP, IMP, inosine, and hypoxanthine. The ATP has no taste, but it is already known that AMP brings the meat flavor sweeter when it is at the level of 5–100 mg/100 mL. At this level, IMP has the ability to combine with umami flavor in a synergistic manner [50]. Inosine monophosphate (IMP) is well-known for its strong umami and salty flavor, which is usually more intense than the monosodium glutamate (MSG) flavor [50] and is very important to the flavor of chicken meat [30]. The delighted products of ATP, especially IMP and GMP, add to the enhanced umami flavor, especially in a synergistic reaction with L-glutamate (L-Glu), which augments the perception of the salty flavor [58]. Fermented goji berry residue dietary supplementation had significant positive endpoints of inosine monophosphate (IMP) and glutamate in the LD (*p* < 0.05). The IMP showed significant flavor-modifying effects, especially by umami potentiation and palatability enhancement. The FGB group had better levels of IMP compared to the controls, and L-glutamate (L-Glu) had a synergistic effect on the enhancement of flavor profile in Tan sheep meat. It is worth mentioning that the degradation products of IMP (inosine and hypoxanthine) were found to be the sources of bitter taste [59]. The FGB group exhibited significantly reduced hypoxanthine concentrations compared to controls (*p* < 0.001). Such a low concentration indicates that fermented goji berry residues would be beneficial to the meat, reducing bitter-tasting molecules, enhancing the meat flavor, and generating a softer taste profile. Primary contributors to food sourness are organic acids, with lactic acid being the major one. Lactic acid is produced during postmortem glycolysis [60], and research shows that the level of lactic acid is positively related to the sourness of the Choshu-Kurokashiwa chicken broth, and thus, the higher the level of lactic acid, the higher the sourness of the chicken broth [61]. The recorded increase in lactic acid is probably a result of the microbial metabolic processes during the fermentation process. Lactic acid, being one of the major organic acids in meat products, adds to both the sour taste and flavor complexity. These results indicate that fermented goji berry residual supplementation improves Tan sheep meat flavor by coordinated regulation of lactic acid, IMP, and hypoxanthine concentrations that eventually maximize the sensory quality parameters. Fermented goji berry residue as a natural feed additive is capable of improving the quality of the flavor of Tan sheep meat, improving the levels of umami and decreasing the levels of bitterness, which has given promising prospects of application in the livestock production and the meat processing industry.

### 4.4. Impact of FGB on Fatty Acid Profiling in Longissimus Dorsi Muscle of Finishing Tan Sheep

Palmitic, oleic, and stearic acids were the prevalent ones in CON and FGB groups, as in Tan sheep profiles [45,62]. Total SFAs were significantly reduced by FGB supplementation compared to CON (*p* < 0.05), with a significant reduction in atherogenic myristic (C14:0) and palmitic acids (C16:0)—major contributors of LDL-cholesterol deposition and cardiovascular risk [63]. This adjustment implies that FGB gives a healthier lipid profile. Importantly, insulin resistance is stimulated by palmitic acid (28–32% of circulating FFAs) through FOXO1 signaling, which is one of the central pathways in type 2 diabetes etiology [64]. Activating the FOXO1-related pathways, palmitic acid is one of the key factors that hasten the onset of insulin resistance, which is one of the major processes of metabolic homeostasis. Also, research has shown that some amino acids, including leucine and valine, have the ability to inhibit gluconeogenesis in the liver and dorsal fat through serine/threonine protein kinase (AKT)/FOXO1 signaling pathway and, thus, restore glucose homeostasis. This is a saturated fatty acid-sensitive pathway; high saturated fat diets would increase the dysregulation of AKT/FOXO1 and disrupt glucose metabolism [65]. These results suggest that fermented goji-berry pomace may lower saturated fatty acid content in the *Longissimus dorsi* by modulating these FOXO1-mediated pathways.

Dietary FGB supplementation significantly decreased key MUFAs/PUFAs (C17:1n7, oleic acid, trans-vaccenic acid, linoleic acid, arachidonic acid) while increasing heptadecanoic (C17:0) and stearic acid (C18:0) in *Longissimus dorsi* versus CON (*p* < 0.05). Transcriptomic profiling identified *FADS1* and *FADS2* as enriched in fatty acid metabolic pathways, with *FADS2* additionally enriched in the α-linolenic acid metabolism pathway; both genes were downregulated in the FGB group. Previous reports have established that *FADS1*, *FADS2*, and *FADS3* orchestrate the biosynthesis of highly unsaturated fatty acids, and that *FADS2*’s multifunctional desaturase activity (Δ6, Δ8, Δ4) is critical for the generation of various unsaturated fatty acids, including arachidonic acid [66]. It catalyzes the introduction of an n–11 double bond in odd-chain monounsaturated fatty acids and n–10 and n–12 double bonds in even-chain monounsaturated fatty acids [67]. Based on this evidence, we hypothesize that fermented goji berry residue induces differences in unsaturated fatty acid content within the *Longissimus dorsi* muscle by modulating fatty acid metabolism and alpha-linolenic acid metabolism, mediated through *FADS1* and *FADS2*. Oleic acid, a key contributor to meat palatability and aroma [68], was the predominant fatty acid in both groups. The observed differences in levels of cis-C18:1n9 (oleic acid) suggest that the addition of fermented goji berry residue may influence the flavor characteristics of the *longissimus dorsi* muscle in Tan sheep. Furthermore, higher levels of arachidonic acid are associated with enhanced umami perception [69], and significant intergroup differences in arachidonic acid content may help explain the distinctive flavor profile of Tan sheep meat. Fermented goji pomace supplementation promotes branched-chain fatty acid (BCFA) biosynthesis—key contributors to lamb’s distinctive flavor [70]—while remodeling the fatty acid profile of Tan sheep meat through reduced SFAs and elevated UFAs/BCFAs. These shifts collectively enhance both nutritional and flavor attributes.

### 4.5. Impact of FGB on Volatile Flavor Compounds in Longissimus Dorsi Muscle of Finishing Tan Sheep

A total of 13 compounds were detected in both the FGB and CON groups: Carbon disulfide (sweet, pleasing, ethereal odor), 2-Butanone (acetone-like odor, sweet, pleasant, pungent), 1-Butanol (rancid, sweet, strong characteristic, mildly alcoholic odor), Decane (alkane), Acetone (fruity odor, characteristic odor, pungent, sweetish), Acetic acid (pungent, sour, vinegar-like odor, burning taste, sharp), Phenylacetaldehyde (bitter, clover, cocoa, floral, grapefruit, green, hawthorn, honey, hyacinth, peanut), Octane (gasoline-like, alkane), and compounds with unknown aroma characteristics (Benzene, 1,4-bis(1,1-dimethylethyl)-; Heptane, 4-methyl-; 2,4-Dimethyl-1-heptene; Hydroxylamine, O-(2-methylpropyl)-; 1,3,5-Cycloheptatriene). These compounds can be considered typical “Tan sheep meat flavor” compounds.

Volatile compound analysis revealed significantly elevated 1-Butanol and Carbon disulfide in FGB-supplemented meat (*p* < 0.05), with acetic acid showing an increasing trend. Additionally, 16 unique flavor compounds were exclusively detected in the FGB group (Table 8), collectively indicating substantial modification of Tan sheep meat’s sensory profile. While alcohol levels (notably 1-Butanol) were significantly elevated in FGB-supplemented meat—likely derived from lipid/protein degradation [71]—their high odor thresholds limit aromatic contributions. Such changes in composition can contribute to flavor mildly, without adding much to aroma. Aldehydes and other compounds that produce meat flavor are primarily products of fat oxidation and degradation. Aldehydes are characterized by low odor thresholds and are reputed to cause greasy and off-flavors in meat [72]. A majority of straight-chain aldehydes have an unpleasant smell, mainly because of the oxidation of unsaturated fatty acids. At low aldehyde concentrations, their typical odors, especially unsaturated aldehyde, may come out, which is relevant to taste. FGB supplementation lowered total aldehydes significantly compared to CON (*p* < 0.05) and also significantly decreased phenylacetaldehyde, a Strecker aldehyde of methionine/phenylalanine degradation related to beer spoilage [73] and beer off-flavors [74]. This is a likely minimization of off-flavor possibilities in *Longissimus dorsi*.

Proteins play a key role in binding flavor compounds as an essential matrix, due to the flavor being one of the vital sensory attributes [54]. This paper demonstrates that when fermented goji berry pomace is added to the diet, the relative levels of the aldehydes decreased. This could be due to an augmentation of the muscle proteins, which eventually affected the liberation of aldehydes [75,76]. Nonetheless, the mediating mechanism of the muscle protein flavor is yet to be explored.

Hydrocarbons are the primary VOCs in the *Longissimus dorsi*, with the highest relative content observed across all samples. These hydrocarbons primarily originate from the cleavage of fatty acid alkoxy radicals. The compounds detected in this study were mainly alkanes and terpenes, which have higher odor thresholds and only a minor direct impact on Tan sheep meat flavor. These compounds function as critical intermediates for flavor-enhancing heterocyclics, while key volatiles (acetic acid, butanoic acid, phenylacetaldehyde, and 3-methylbutanal) identified in Hungarian salami were also detected in our system. The relative content differences in these compounds due to the addition of fermented goji berry pomace may influence the flavor of Tan sheep meat [77].

### 4.6. Integrated Metabolomic and Transcriptomic Analysis of FGB’s Mechanistic Impact on Amino Acid and Lipid Metabolism in Longissimus Dorsi Muscle of Finishing Tan Sheep

The mechanism by which nutritional factors are associated with lamb flavor through their impact on muscle metabolism may be related to gene regulation to some extent. Therefore, integrating transcriptomics and metabolomics analyses provides a powerful means to elucidate the regulatory mechanisms of meat quality formation in livestock and poultry [78]. Our study reveals that fermented goji pomace modulates Tan sheep meat quality parameters by regulating amino acid and lipid metabolism pathways, thereby enhancing our understanding of molecular-level quality control mechanisms. KEGG enrichment analysis of metabolomic profiles between the LDM6 and LDMCON6 groups revealed significant overrepresentation of differential metabolites in pathways including butanoate metabolism, glycolysis/gluconeogenesis, PI3K-Akt signaling, HIF-1 signaling, alcoholic liver disease, pyruvate metabolism, and glyoxylate and dicarboxylate metabolism (Figure 2F,G). Taken together, the pathways of the enriched terms are mainly associated with amino acid metabolism and lipid metabolism, indicating that changes in the biochemical processes are major causes of the observed discrepancies in the *longissimus dorsi* muscle quality and flavor in Tan sheep. Transcriptomic analyses of the LD revealed 382 DEGs between the LDM and LDMCON conditions, including 162 upregulated genes and 220 downregulated genes. GO and KEGG enrichment was further conducted on pathways and terms that are relevant to meat quality and flavor to select the most important genes involved in lipid and amino acid metabolism and visualize them in the form of Cytoscape. The degree value was used to rank the genes by their importance in the network, with the core genes shown in dark colors (Figure 4E,F). The inclusion of genes like *FOXO1*, *SLC2A4*, *LPIN1*, *IGF1*, *SPP1*, *COL3A1*, *GLUL*, and *PSMC1* in the middle of the PPI networks implies that they can have important roles in the regulation of the metabolic pathways involved in the determination of muscle quality and flavor.

Regarding lipid metabolism, in mammary epithelial cells, *FOXO1* controls the triglyceride production and lipid droplet generation by controlling the expression of lipid metabolism genes, including *CD36* and *STEAP4*. In hepatocytes, *FOXO1* deficiency results in decreased expression of fatty acid oxidation-related genes, including *CPT1α* and *ATGL*, which exacerbates lipid accumulation [79]. *FOXO1* regulates lipogenesis via the AMPK and PI3K/AKT signaling pathways. *SLC2A4* (solute carrier family 2 member 4) has been shown to negatively regulate the deposition of lipids in pigs and sheep. Its expression can be increased, which stimulates the supply of energy to adipocytes due to the enhancement of the transport of glucose, which triggers lipogenesis [80]. Furthermore, *SLC2A4* can be involved in fatty acid oxidation and energy metabolism in the thermogenic pathways of sheep, including other related genes like *ACSL1* and *CPT1A* [81]. In addition, *SLC2A4* has the potential to affect lipid homeostasis by regulating transmembrane fatty acid transport, possibly via miR-345-3p interaction. The role of its action is also linked to the insulin signaling pathway and the AMPK pathway, which is also a crucial element in fat deposition in sheep [80,82]. As an example, the decreased AMPK activity can mediate fat deposition in the growth of sheep by the *SLC2A4*, and the fatty acid degradation and triglyceride metabolism may be mainly regulated by *FOXO1* and *SLC2A4*, respectively [83]. *LPIN1* is particularly associated with triglyceride metabolism and fatty acid degradation. It has been established that the expression of *IGF1* is significantly enhanced in the fat tissue of obese individuals, including human and animal subjects. This increase is associated with increased lipogenic enzyme activity and is capable of promoting adipose deposition by the activation of the PI3K-AKT/AMPK signaling pathway [16]. Thus, the feeding of fermented goji berry residues can have an impact on intramuscular fat levels and, consequently, on the nutritional and sensorial properties of Tan sheep meat through the regulation of the expression of critical genes (*FOXO1*, *SLC2A4*, *LPIN1*, *IGF1*).

Regarding the metabolism of the amino acids, COL3A1 (type III collagen) is of paramount importance to collagen synthesis, which is strongly connected to the requirement of certain amino acids, including proline and glycine. It has been found that PDHPS1 modulates the expression of *COL3A1*, which affects the amino acid synthesis-related metabolic processes by inhibiting the *PKM2* (pyruvate kinase M2) expression and the Smad 2 phosphorylation. The modulation, in turn, affects the synthesis of glycolytic intermediates such as glucose and pyruvate [84]. GLUL (glutamine synthetase) is an important enzyme that is vital in the glutamate–glutamine metabolism and the establishment of amino acid homeostasis. The studies have shown that *FOXO1* knockout could revert BaP-induced changes in glutamate–glutamine metabolism by increasing GLUL and SLC1A3, which emphasizes the repair role played by GLUL in amino acid metabolism [85]. Moreover, it has been revealed that hepatic *GLUL* mRNA expression among obese individuals positively correlates with the severity of MASLD and is accompanied by an increase in the levels of glutamate in the bloodstream, which indicates that *GLUL* can also mediate the amino acid metabolism via the nitrogen metabolic imbalance [86]. In another study, it was found that N-carbamylglutamate (NCG) supplementation had a significant effect on the ruminal fluid of sheep, mainly in glutamate- and nitrogen-related metabolites, suggesting that GLUL-related pathways could successfully be manipulated in sheep amino acid metabolism [87]. In addition, *GLUL* is a differentially expressed gene related to muscle growth in sheep, where its expression could be associated with the metabolic path of alanine, aspartate, and glutamate [88]. *PSMC1* (proteasome 26S subunit ATPase) is an integral component of the proteasome system, involved in protein degradation and amino acid recycling. Additionally, endurance exercise may impact amino acid metabolism by altering protein degradation, potentially involving proteasomal components like *PSMC1*. Thus, *PSMC1* may indirectly influence the dynamic balance of the free amino acid pool by regulating the rate of protein degradation [89]. In summary, *COL3A1* regulates the distribution of specific amino acids (e.g., glycine and proline) through collagen metabolic demand and coordinates with glycolysis–TCA cycle metabolism [84,90]. GLUL, as a rate-limiting enzyme in glutamine synthesis, modulates nitrogen metabolism and the glutamate/glutamine ratio, influencing amino acid homeostasis in the liver and muscle [87,91]. Meanwhile, *PSMC1* may regulate protein degradation via proteasomal activity, releasing free amino acids for metabolism or new protein synthesis [89,92]. Therefore, feeding fermented wolfberry pomace may influence muscle development and protein deposition in muscles by regulating the expression of key genes *(COL3A1*, *GLUL*, *PSMC1*), thereby improving the nutritional and flavor quality of Tan sheep meat.

The integration of transcriptomic and metabolomic data provides a powerful approach for understanding the molecular mechanisms underlying lamb meat quality attributes. Notably, the enriched KEGG pathways identified from differentially expressed genes (DEGs) and differentially accumulated metabolites (DMs) showed significant overlap. Specifically, 42 KEGG pathways were found to be concurrently enriched by both DEGs and DMs, as shown in Figure 6A. To visualize the relationships between genes, KEGG pathways, and metabolites, we employed a Sankey diagram to illustrate the significantly enriched KEGG pathways (Figure 6B). These nine key KEGG pathways involved a total of 31 differentially expressed genes, including *GLUL*, *COL21A1*, *COL6A6*, *COL15A1*, *COL8A1*, *COL3A1*, *IL6R*, *IGF2*, *SPP1*, *IGF1*, *ITGA7*, *PFKFB3*, *FOXO1*, *FAS*, and *SLC2A4*, which are closely associated with important biological function metabolites such as nitric oxide, acetic acid, 2-butenedioic acid (2-methyl-, (E)-), and ethanol.

These genes are closely associated with several important biofunctional metabolites such as nitric oxide, acetic acid, 2-butenedioic acid (2-methyl-, (E)-), and ethanol. Based on these findings, our study reveals that fermented goji pomace likely enhances Tan lamb meat quality and flavor attributes by modulating amino acid and lipid metabolism pathways. Key metabolites involved include nitric oxide, acetic acid, (E)-2-methyl-2-butenedioic acid, and ethanol. Through functional enrichment and protein–protein interaction (PPI) network analyses, we identified critical regulatory genes strongly associated with flavor-related amino acids (*COL3A1*, *GLUL*, *PSMC1*) and lipid metabolism (*FOXO1*, *SLC2A4*, *LPIN1*, *IGF1*, *SPP1*).

Overall, our results support the potential of fermented Goji berry residue (FGB) as a value-added feed ingredient for finishing Tan sheep, linking improvements in meat nutritional traits and flavor-related indicators with coordinated changes in amino acid and lipid metabolism. From a practical standpoint, this strategy may provide a feasible route for the upcycling of goji-processing by-products in Ningxia, thereby reducing waste-disposal pressure while contributing to premium lamb production. For industry translation, key issues that warrant further evaluation include (i) cost-effectiveness relative to conventional ingredients (including fermentation, drying, and logistics), (ii) scalability and quality control of fermentation (batch-to-batch consistency of bioactive components and nutrient composition), (iii) formulation compatibility and on-farm performance across production systems (palatability, intake, growth, carcass grading, and health outcomes), and (iv) sensory validation in consumer panels to confirm whether the observed biochemical changes translate into perceivable improvements in eating quality. Addressing these points in future trials will help determine the most appropriate inclusion level, feeding duration, and processing specifications for commercial application.

## 5. Conclusions

In summary, supplementing the diet of fattening Tan sheep with 4% fermented goji pomace improved meat quality and flavor-related traits, with the *Longissimus dorsi* (LD) as the primary muscle investigated for multi-omics analyses. This dietary addition significantly increased the brightness (L*) and redness (a*) of the LD muscle, while increasing crude protein content and reducing crude fat content. Flavor precursor profiles were also favorably shifted, as reflected by increased concentrations of several amino acids (e.g., Lys, Met, and His) and higher levels of lactic acid and inosine monophosphate (IMP), together with reduced hypoxanthine. Untargeted GC-TOFMS metabolomics and RNA-seq transcriptomics in LD identified distinct metabolic and transcriptional responses to FGB supplementation, with differential features enriched in pathways related to energy metabolism and nutrient (amino acid/lipid) metabolism. Key candidate regulatory genes associated with lipid- and amino acid-related processes were also identified, providing a molecular basis for future mechanistic verification.

However, meat quality parameters varied among the four muscles studied, and the effects of fermented goji pomace were muscle-specific. These findings suggest that the metabolic and gene regulatory responses to dietary supplementation may differ across muscle types. Further studies are needed to explore these differences in additional muscles and under commercial production conditions and to confirm mechanistic links using targeted functional validation.

## Figures and Tables

**Table 1:** Composition and nutrient levels of basal diets (%, dry matter basis).

Items	CON	FGB
Ingredient		
Corn	30.0	26.0
Finishing sheep total mixed diet ^(1)^	20.0	20.0
Silage corn	10.0	10.0
Corn straw	20.0	20.0
Fattening feed ^(2)^	20.0	20.0
Fermented goji berry residue	/	4.00
Total	100.0	100.0
Chemical composition		
Metabolizable energy (ME)/(MJ/kg) ^(3)^	9.30	9.16
Crude protein (CP)	13.52	15.01
Neutral detergent fiber (NDF)	32.48	32.97
Acid detergent fiber (ADF)	45.73	47.45
Ca	0.56	0.57
P	0.38	0.41

Note: ^(1)^ Composition content of finishing sheep total mixed diet: CP ≥ 15.0%, CF ≤ 20.0%, Ash ≤ 13.5%, moisture ≤ 13.0%, Lys ≥ 0.6%, NaCl 0.5~1.0%, Ca 0.5~1.5%, *p* ≥ 0.4%; ^(2)^ Fattening feed: CP ≥ 35.0%, CF ≤ 14.0%, Ash ≤ 20.0%, moisture ≤ 14.0%, Lys ≥ 1.1%, NaCl 2.5~5.0%, Ca 2.0~5.0%, *p* ≥ 0.5%; ^(1,2)^ All are complete formula feed; ^(3)^ ME was a calculated value, while the others were measured values.

**Table 2:** Nutrient levels of goji berry residue before and after fermentation (%, dry matter basis).

Chemical Composition	Unfermented	Fermented
Dry matter	36.78	38.31
Crude protein	8.56	9.77
Crude fat	6.52	7.03
Soluble carbohydrates	0.31	0.47
Polysaccharide	1.03	1.65
Neutral detergent fiber	71.34	63.31
Acid detergent fiber	64.62	55.37

**Table 3:** Effects of fermented goji berry residue on the meat quality in LD, TM, GM, and BF muscles of Tan sheep.

Items	Muscles	CON	FGB	*p*-Value
pH_45min_	TM	6.34 ± 0.13	6.52 ± 0.31	0.412
	GM	6.46 ± 0.20	6.16 ± 0.58	0.441
	LD	6.45 ± 0.11	6.17 ± 0.17	0.071
	BF	6.42 ± 0.45	6.41 ± 0.63	0.977
pH_24h_	TM	5.93 ± 0.11	6.19 ± 0.30	0.235
	GM	6.08 ± 0.20	5.72 ± 0.60	0.383
	LD	6.08 ± 0.09	5.87 ± 0.26	0.259
	BF	6.06 ± 0.45	6.02 ± 0.65	0.933
L* (Lightness)	TM	30.8 ± 0.18 ^b^	35.8 ± 0.12 ^a^	<0.001
	GM	28.5 ± 0.15 ^b^	38.1 ± 0.03 ^a^	<0.001
	LD	29.1 ± 0.21 ^b^	31.2 ± 0.22 ^a^	<0.001
	BF	30.1 ± 0.37 ^a^	27.4 ± 0.11 ^b^	<0.001
a* (Redness)	TM	23.8 ± 1.60	21.7 ± 0.28	0.144
	GM	24.7 ± 0.22	33.3 ± 5.78	0.125
	LD	18.0 ± 0.16 ^b^	23.2 ± 0.48 ^a^	<0.001
	BF	20.1 ± 0.97	19.6 ± 0.50	0.460
b* (Yellowness)	TM	1.8 ± 0.17 ^b^	2.7 ± 0.16 ^a^	0.002
	GM	3.8 ± 0.21 ^a^	3.1 ± 0.14 ^b^	0.013
	LD	3.0 ± 0.04	3.0 ± 0.01	0.783
	BF	2.3 ± 0.36 ^b^	3.5 ± 0.23 ^a^	0.009
Drip loss (%)	TM	4.63 ± 2.03	4.56 ± 0.7	0.960
	GM	6.51 ± 1.31	6.33 ± 2.09	0.904
	LD	5.29 ± 2.71	5.01 ± 0.82	0.872
	BF	0.94 ± 0.03	0.94 ± 0.04	0.7726
Cooking loss (%)	TM	23.6 ± 4.87	20.63 ± 6.14	0.548
	GM	19.09 ± 5.32	20.61 ± 9.76	0.824
	LD	12.1 ± 5.63	20.07 ± 8.94	0.262
	BF	19.37 ± 5.69	20.13 ± 8.24	0.901
Shear force (N)	TM	13.3 ± 0.49	14.11 ± 0.91	0.247
	GM	21.12 ± 0.96 ^b^	23.83 ± 0.34 ^a^	0.010
	LD	15.04 ± 1.39	14.34 ± 0.21	0.440
	BF	17.36 ± 0.85	18.74 ± 0.7	0.096

Note: Trial group = Fermented goji berry residue; Con group = Control group (n = 3, three lambs per replicate/pen). ^a,b^ Means with different superscripts within the same row are different from each other (*p* < 0.05).

**Table 4:** Effects of fermented goji berry residue on the muscle chemical composition in LD, TM, GM, and BF muscles of Tan sheep.

Items	Muscles	CON	FGB	*p*-Value
Moisture (%)	TM	77.14 ± 0.58	77.79 ± 0.30	0.159
	GM	76.64 ± 0.57	76.92 ± 0.44	0.539
	LD	74.12 ± 0.81	74.89 ± 0.53	0.238
	BF	75.45 ± 0.44	75.84 ± 0.50	0.368
Ash content (%)	TM	1.14 ± 0.02	1.21 ± 0.05	0.069
	GM	1.76 ± 0.05	1.84 ± 0.07	0.187
	LD	1.12 ± 0.04	1.17 ± 0.04	0.183
	BF	1.11 ± 0.11	1.16 ± 0.04	0.499
Crude protein (%)	TM	8.18 ± 0.10 ^a^	6.11 ± 0.02 ^b^	<0.001
	GM	7.84 ± 0.06 ^b^	8.67 ± 0.18 ^a^	0.002
	LD	7.05 ± 0.11 ^b^	8.06 ± 0.15 ^a^	0.001
	BF	7.94 ± 0.11 ^b^	8.54 ± 0.06 ^a^	0.001
Crude fat (%)	TM	11.76 ± 0.33 ^a^	9.04 ± 0.31 ^b^	<0.001
	GM	18.33 ± 0.24 ^a^	13.63 ± 0.48 ^b^	<0.001
	LD	15.25 ± 0.3 ^a^	7.66 ± 0.10 ^b^	<0.001
	BF	12.74 ± 0.54 ^a^	4.72 ± 0.25 ^b^	<0.001

Note: Trial group = Fermented goji berry residue; Con group = Control group (n = 3, three lambs per replicate/pen). ^a,b^ Means with different superscripts within the same row are different from each other (*p* < 0.05).

**Table 5:** Effects of fermented goji berry residue on the amino acid contents in LD muscle of Tan sheep (μg/g).

Items	CON	FGB	*p*-Value
Essential amino acid (EAA)			
Lysine (Lys)	23,352.97 ± 382.40 ^b^	24,230.2 ± 365.50 ^a^	0.045
Phenylalanine (Phe)	8424.78 ± 182.61	8719 ± 137.87	0.090
Methionine (Met)	4561.85 ± 119.43 ^b^	4973.28 ± 46.51 ^a^	0.005
Threonine (Thr)	9962.52 ± 164.97	10,420.53 ± 193.19	0.073
Valine (Val)	9701.99 ± 209.02	9900.14 ± 199.99	0.301
Leucine (Leu)	17,275.44 ± 362.4	17,787.52 ± 280.94	0.125
Isoleucine (Ile)	9663.32 ± 199.52	9856.2 ± 181.18	0.283
Histidine (His)	6992.52 ± 151.5 ^b^	7528.72 ± 130.59 ^a^	0.044
Total EAA ^a^	89,935.38 ± 1718.66	93,115.59 ± 1503.02	0.073
Non-essential amino acid (NEAA)			
Asparagic acid (Asp)	22,022.31 ± 512.74	22,772.96 ± 629.23	0.184
Glutamic acid (Glu)	35,843.9 ± 829.82	37,386.5 ± 929.49	0.099
Glycine (Gly)	9054.85 ± 178.3 ^b^	9640.38 ± 145.7 ^a^	0.012
Alanine (Ala)	12,464.31 ± 240.09	12,874.62 ± 234.52	0.102
Proline (Pro)	8014.56 ± 157.71 ^b^	8461.58 ± 117.48 ^a^	0.017
Arginine (Arg)	13,415.12 ± 201.4 ^b^	14,355.53 ± 332.51 ^a^	0.014
Serine (Ser)	8666.07 ± 128.75	8980.65 ± 190.38	0.077
Tyrosine (Tyr)	7289.04 ± 150.44	7550.37 ± 115.19	0.075
Cysteine (Cys)	292.34 ± 21.37 ^b^	348.34 ± 16.11 ^a^	0.022
Total amino acid	199,464.65 ± 9710.99	215,486.51 ± 3999.45	0.057
Total NEAA ^b^	109,529.27 ± 10,847.73	122,370.93 ± 2537.76	0.117
EAA/NEAA (%)	82.76 ± 0.94	76.1 ± 0.53	0.311
Total flavor amino acids ^c^	92,800.49 ± 1949.27	97,029.99 ± 2184.47	0.067
Total sweet amino acids (SAAs) ^d^	48,162.31 ± 864.85 ^b^	50,277.75 ± 823.98 ^a^	0.037
Total bitter amino acids (BAAs) ^e^	61,610.24 ± 1241.31	64,201.39 ± 1153.24	0.057

Note: ^a^ EAA = Lys + Phe + Met + Thr + Val + Leu + Ile + His. ^b^ NEAA = Asp + Glu + Gly + Ala + Pro + Arg + Ser + Tyr + Cys. ^c^ Flavor amino acid = Glu + Asp + Ala + Arg + Gly. ^d^ SAA= Ala + Gly + Pro + Ser + Thr. ^e^ BAA = Arg + His + Ile + Leu + Met + Val. Results are presented as the mean ± SE (n = 6).

**Table 6:** Effects of fermented goji berry residue on the contents of meat flavor compounds in LD muscle of Tan sheep (μg/g).

Items	CON	FGB	*p*-Value
Lactic acid	2932.24 ± 856.96 ^b^	4595.45 ± 335.38 ^a^	0.004
Fumaric acid	304.54 ± 77.82	308.2 ± 17.33	0.914
Succinic acid	2378.5 ± 125.09	2030.17 ± 428.55	0.085
Inosine monophosphate (IMP)	0.62 ± 0.02 ^b^	0.8 ± 0.13 ^a^	0.017
Hypoxanthine	764.2 ± 2.71 ^a^	718.8 ± 1.47 ^b^	<0.001

Note: Results are presented as the mean ± SE (n = 6). ^a,b^ Means with different superscripts within the same row are different from each other (*p* < 0.05).

**Table 7:** Effects of fermented goji berry residue on the fatty acid composition in LD muscle of Tan sheep (mg/kg).

Items	CON	FGB	*p*-Value
C10:0	25.72 ± 1.72	25.75 ± 0.85	0.982
C12:0	35.47 ± 1.17	36.37 ± 0.63	0.309
C14:0	417.47 ± 12.94 ^a^	371.11 ± 18.63 ^b^	0.024
C15:0	69.79 ± 1.96 ^b^	89.91 ± 6.93 ^a^	0.008
C16:0	2900.66 ± 18.03 ^a^	2221.93 ± 121.39 ^b^	0.009
C17:0	139.62 ± 9.75 ^b^	225.08 ± 5.53 ^a^	<0.001
C18:0	1477.99 ± 25.15 ^b^	1790 ± 42.54 ^a^	<0.001
iso-C14:0	14.18 ± 0.36 ^b^	16.45 ± 1.36 ^a^	0.049
anteiso-C14:0	13.89 ± 0.13 ^b^	22.32 ± 0.81 ^a^	0.003
iso-C15:0	33.51 ± 0.99	35.99 ± 2.7	0.209
anteiso-C16:0	106.76 ± 2.33 ^b^	120.82 ± 3.91 ^a^	0.006
C16:1n9	90.6 ± 1.14	86.24 ± 3.19	0.090
C16:1n7	344.08 ± 28.74 ^a^	264.99 ± 12.48 ^b^	0.012
C17:1n7	112.06 ± 2.15 ^a^	92.88 ± 5.2 ^b^	0.004
C18:1n9 cis	2522.43 ± 44.29 ^a^	2176.86 ± 72.72 ^b^	0.002
C18:1n9 trans	97.34 ± 0.79 ^a^	88.77 ± 3.3 ^b^	0.040
C18:1n7	30.91 ± 0.49 ^b^	43.09 ± 0.95 ^a^	<0.001
C18:1n5	28.92 ± 0.62	28.75 ± 0.94	0.803
C18:2n6 (LA)	314.99 ± 7.03 ^a^	230.54 ± 11.62 ^b^	<0.001
C19:1n9 cis	19.22 ± 0.8 ^b^	22.16 ± 0.82 ^a^	0.011
C18:2n7 (CLA)	22.73 ± 0.59 ^b^	59.54 ± 5.56 ^a^	0.007
C20:4n6 (ARA)	89.48 ± 5.31 ^a^	57.4 ± 1.22 ^b^	0.007
SFA ^a^	5235.07 ± 52.51	4955.73 ± 201.96	0.081
BCFA ^b^	163.58 ± 5.17 ^b^	195.57 ± 8.62 ^a^	0.005
MUFA ^c^	3245.58 ± 56.48 ^a^	2803.74 ± 96.5 ^b^	0.002
PUFA ^d^	427.2 ± 6.04 ^a^	347.48 ± 15.26 ^b^	0.001
ω-6 PUFA ^e^	404.46 ± 5.52 ^a^	287.94 ± 12.81 ^b^	<0.001
PUFA/SFA (%)	8.16 ± 0.04 ^a^	7.01 ± 0.08 ^b^	<0.001

Note: ^a^ SFA = C10:0 + C12:0 + C14:0 + C15:0 + C16:0 + C17:0 + C18:0 + iso-C14:0 + anteiso-C14:0 + iso-C15:0 + anteiso-C16:0. ^b^ BCFA = iso-C14:0 + anteiso-C14:0 + iso-C15:0 + anteiso-C16:0. ^c^ MUFA = C16:1n9 + C16:1n7 + C17:1n7 + C18:1n9 cis + C18:1n9 trans + C18:1n7 + C18:1n5 + C19:1n9 cis. ^d^ PUFA = C18:2n6 + C18:2n7 + C20:4n6. ^e^ ω-6 PUFA = C18:2n6 + C20:4n6. Results are presented as the mean ± SE (n = 6).

**Table 8:** Effects of fermented goji berry residue on the volatile compounds in LD muscle of Tan sheep.

Volatiles	Odor Description *	Relative Peak Area of Total Peak (%)		*p*-Value
		CON	FGB	
Alcohols				
1-Butanol	Rancid, sweet, strong characteristic, mildly alcoholic odor ^1^; fruit, medicine ^2^	3.77 ± 1.39 ^b^	21.13 ± 1.83 ^a^	<0.001
1-Pentanol, 2-methyl-	NA	0.9 ± 0.19	nd.	-
Aldehydes				
Phenylacetaldehyde	Bitter, clover, cocoa, floral, grapefruit, green, hawthorne, honey, hyacinth, peanut ^2^	3.93 ± 0.62 ^a^	0.94 ± 0.4 ^b^	0.002
Methacrolein	NA	nd.	0.5 ± 0.12	-
Butanal, 3-methyl-	Apple-like odor, acrid odor, warm, herbaceous, slightly fruity, and nut-like ^1^	6.53 ± 2.95	nd.	-
Ketones				
2-Butanone	Acetone-like odor, sweet, pleasant, pungent ^1^; acetone, camphor, ether, ethereal, fruity ^2^	10.91 ± 1.87	16.49 ± 5.9	0.193
Acetone	Fruity odor, characteristic odor, pungent, sweetish ^1^; apple, ethereal, pear, solvent ^2^	1.07 ± 0.43	6.2 ± 7.56	0.36
p-Pentylacetophenone	NA	0.47 ± 0.27	nd.	-
Hydrocarbons				
Decane	Alkane ^2^	8.48 ± 0.96 ^a^	5.82 ± 1.05 ^b^	0.032
1-Hexene, 4,5-dimethyl-	NA	nd.	1.38 ± 0.22	-
1-Fluorooctane	NA	nd.	1.27 ± 0.49	-
Benzene, 1,4-bis(1,1-dimethylethyl)-	NA	1.59 ± 0.72	2.96 ± 1.76	0.281
Heptane, 4-methyl-	NA	3.05 ± 0.07	3.27 ± 1.06	0.756
2,4-Dimethyl-1-heptene	NA	14.6 ± 0.81	15.17 ± 2.22	0.7
Oxirane, pentyl-	NA	nd.	0.6 ± 0.19	-
Hexane, 2,3-dimethyl-	NA	nd.	4.1 ± 1.34	-
1,3,5-Cycloheptatriene	NA	0.56 ± 0.04 ^a^	0.34 ± 0.04 ^b^	0.002
Octane	Gasoline-like ^1^; alkane ^2^	19.38 ± 0.28	17.36 ± 7.23	0.676
2-Pentene	NA	nd.	1.03 ± 0.33	-
1,6-Heptadien-3-yne	NA	nd.	0.6 ± 0.06	-
2,4,6-Trimethyl-1-nonene	NA	1.55 ± 1.06	nd.	-
Octane, 2-methyl-	NA	4.93 ± 1.46	nd.	-
Hexane, 3,3-dimethyl-	NA	1.72 ± 0.22	nd.	-
Hexane, 2,3,5-trimethyl-	NA	1.38 ± 0.36	nd.	-
Hexane, 2,4-dimethyl-	NA	0.52 ± 0.1	nd.	-
1-Hexene, 3,3-dimethyl-	NA	3.34 ± 0.93	nd.	-
Dodecane	Alkane ^2^	7.46 ± 4.17	nd.	-
Benzene, 1,3-bis(1,1-dimethylethyl)-	NA	1.26 ± 0.83	nd.	-
Acetic acid	Pungent, sour, vinegar-like odor, burning taste ^1^; sharp ^2^	6.01 ± 0.83	11.09 ± 3.14	0.099
1H-Indene-4-carboxylic acid, 2,3-dihydro-1,1-dimethyl-	NA	nd.	2.72 ± 2.26	-
Butanoic acid	Unpleasant, rancid, penetrating and obnoxious odor, butter-fat taste ^1^; acetic, butter, cheese, fruit, sharp, sweet ^2^	20.71 ± 7.62	nd.	-
Esters	NA			
Oxalic acid, allyl nonyl ester	NA	nd.	3.73 ± 1.86	-
Carbamodithioic acid, diethyl-, methyl ester	NA	nd.	4.56 ± 4.88	-
Butanoic acid, ethyl ester	Pineapple odor, sweet ^1^	nd.	8.29 ± 11.94	-
Oxalic acid, isobutyl pentyl ester	NA	nd.	0.89 ± 0.38	-
Ethyl acetate	Fragrant odor, ether-like, fruity odor, fruity with a brandy note ^1^, anise, balsam, green, pineapple, sweet, weedy ^2^	1.3 ± 0.44	nd.	-
Amines				
Hydroxylamine, O-(2-methylpropyl)-	NA	1.99 ± 1.22	0.86 ± 0.34	0.199
2-Propenamide	Odorless ^1^	nd.	0.49 ± 0.12	-
Ethanamine, N-methyl-	NA	nd.	3.06 ± 1.86	-
Acetamide	Mousy odor, a bitter taste ^1^	0.98 ± 0.65	nd.	-
Others				
Carbon disulfide	Sweet, pleasing, ethereal odor ^1^	7.52 ± 0.58 ^b^	15.79 ± 1.49 ^a^	0.001
Acetic acid, lithium salt	NA	nd.	5.51 ± 2.91	-
6H-[1,2,4]Triazolo[1,5-a]indole, 4a,5,7,8,8a,9-hexahydro-9-methylene-	NA	nd.	0.41 ± 0.07	-
Propane, 2-ethoxy-2-methyl-	NA	3.97 ± 3.34	nd.	-

* Flavor description sourced from database available on the web at https://pubchem.ncbi.nlm.nih.gov/ ^1^ and Shin et al. [30] ^2^ NA: not available, data not reported. nd.: not detected. ^a,b^ Means with different superscripts within the same row are different from each other (*p* < 0.05).

## Data Availability

The original contributions presented in this study are included in the article/Supplementary Material. Further inquiries can be directed to the corresponding author

## Supplementary Materials

The following supporting information can be downloaded at: https://www.mdpi.com/article/10.3390/metabo16010039/s1, Table S1: Effect of fermented goji berry residue on the relative content of volatile compounds in LD of Tan sheep; Table S2: The alignment statistics result with the reference gene for all samples; Table S3: Differentially expressed genes in the LDM vs LDMCON group; Table S4: GO enrichment analysis of the LDM vs LDMCON group; Table S5: KEGG enrichment analysis in the LDM vs LDMCON group; Table S6: GO entries and KEGG pathways associated with lipid metabolism and amino acid synthesis.
