# Supplementary material for: Metabolomics and Transcriptomics Reveal the Effects of Fermented Lycium barbarum (Goji) Berry Residue on Muscle Nutrition and Flavor Quality in Fattening Tan Sheep
Source: Metabolites. 2026 Jan 1;16(1):39. doi: 10.3390/metabo16010039 (PMC12843761; doi:10.3390/metabo16010039)
Supplement: Supplementary file 1 [file metabolites-16-00039-s001.zip › Supplementary.pdf]

Supplementary materials

Table S1 Effect of fermented goji berry residue on the relative content of volatile compounds in LD of Tan sheep.

Items	FGB	CON	P
Alcohols	21.13±1.83[a]	4.67±1.2[b]	<0.001
Aldehydes	1.44±0.28[b]	10.47±3.25[a]	0.009
Ketones	22.69±8.44	12.44±1.64	0.166
Hydrocarbons	53.88±10.65	69.83±9.94	0.131
Acids	13.82±1.6[b]	26.72±7.58[a]	0.045
Esters	17.48±18.91	1.3±0.44	0.277
Amines	4.42±2.26	2.97±1.23	0.384
Others	21.71±4.42[a]	11.48±3.15[b]	0.031

Notes: Different superscript letters within each row represent significant differences ($P < 0.05$).

Table S2 The alignment statistics result with the reference gene for all samples.

Samples	Clean reads	Clean bases	GC Content	%≥Q30	Mapped Reads	Uniq Mapped Reads
LDMCON1	38689780	5.8G	52.66	95.32	34285117(88.62%)	31227294(80.71%)
LDMCON2	40671410	6.1G	52.16	95.01	35911948(88.3%)	31307801(76.98%)
LDMCON3	42455094	6.37G	53.49	95.2	37708321(88.82%)	33749670(79.49%)
LDMCON4	39785620	5.97G	53.3	94.98	35385439(88.94%)	32448408(81.56%)
LDMCON5	41579838	6.24G	52.52	95.45	36961838(88.89%)	32695269(78.63%)
LDMCON6	40708542	6.11G	53.78	95.01	35930843(88.26%)	32387550(79.56%)
LDM1	41648550	6.25G	53.31	95.34	37086705(89.05%)	33385199(80.16%)
LDM2	40935234	6.14G	52.82	95.29	36707224(89.67%)	32727995(79.95%)
LDM3	41136666	6.17G	53.61	95.15	36448020(88.6%)	33236403(80.8%)
LDM4	42622594	6.39G	53.44	95.26	37830961(88.76%)	34164716(80.16%)
LDM5	42230064	6.33G	53.62	95.46	37550159(88.92%)	34354193(81.35%)
LDM6	36576664	5.49G	53.19	95.44	32333208(88.4%)	29533169(80.74%)

Notes: Clean reads, the number of clean reads, the single-ended meter; Clean bases, the number of clean data; GC content: the percentage of GC-content in clean data; ≥Q30, Q-score of clean data; ≥30, Mapped reads: the number of reads mapped to the reference genome and its percentage in clean reads; Uniq mapped reads, the number of reads mapped to the only location of the reference genome and its percentage in clean reads. LDM CON, control treatment; LDM, fermented goji berry residue treatment.
